# Supplementary material for: Synthesis, Structural and Physicochemical Characterization of a Titanium(IV) Compound with the Hydroxamate Ligand N,2-Dihydroxybenzamide
Source: Molecules. 2021 Sep 15;26(18):5588. doi: 10.3390/molecules26185588 (PMC8465426; doi:10.3390/molecules26185588)

checkCIF/PLATON report

Structure factors have been supplied for datablock(s) shelx

THIS REPORT IS FOR GUIDANCE ONLY. IF USED AS PART OF A REVIEW PROCEDURE FOR PUBLICATION, IT SHOULD NOT REPLACE THE EXPERTISE OF AN EXPERIENCED CRYSTALLOGRAPHIC REFEREE.

No syntax errors found. CIF dictionary Interpreting this report

Datablock: shelx

```
Bond precision:  C-C = 0.0073 A          Wavelength=0.71073

Cell:             a=20.040(4)      b=26.118(5)      c=36.745(11)
                  alpha=90         beta=90          gamma=90
Temperature:      150 K
```

	Calculated	Reported
Volume	19233(8)	19232(8)
Space group	F d d d	F d d d
Hall group	: -F 2uv 2v	-F 2uv 2v
Moiety formula	C32 H34 K2 N4 O17 Ti2, C H3 O, C O2, 2(C0.50 O0.50), 2(C0.50 O0	C36 H37 K2 N4 O22 Ti2
Sum formula	C36 H37 K2 N4 O22 Ti2	C36 H37 K2 N4 O22 Ti2
Mr	1051.64	1051.69
Dx,g cm-3	1.453	1.453
Z	16	16
Mu (mm-1)	0.587	0.587
F000	8624.0	8624.0
F000’	8644.21	
h,k,lmax	25,32,46	25,32,45
Nref	5004	4976
Tmin,Tmax	0.889,0.943	0.658,0.745
Tmin’	0.889	

```
Correction method= # Reported T Limits: Tmin=0.658 Tmax=0.745
AbsCorr = EMPIRICAL

Data completeness= 0.994          Theta(max)= 26.515

R(reflections)= 0.0718( 3261)     wR2(reflections)= 0.2215( 4976)

S = 1.052                  Npar= 304
```

The following ALERTS were generated. Each ALERT has the format
test-name_ALERT_alert-type_alert-level.
Click on the hyperlinks for more details of the test.

Alert level B

```
PLAT413_ALERT_2_B Short Inter XH3 .. XHn     H3       ..H97C     .       1.97 Ang.
                                                  x,y,z  =      1_555 Check
PLAT430_ALERT_2_B Short Inter D...A Contact  O12      ..O98B     .       2.61 Ang.
                                                  x,y,z  =      1_555 Check
PLAT430_ALERT_2_B Short Inter D...A Contact  O17      ..O98A     .       2.72 Ang.
                                             1/2-x,-y,3/2-z  =     25_556 Check
PLAT430_ALERT_2_B Short Inter D...A Contact  O17      ..O98B     .       2.74 Ang.
                                             1/2-x,-y,3/2-z  =     25_556 Check
PLAT780_ALERT_1_B Coordinates do not Form a Properly Connected Set     Please Do !
```

Alert level C

```
RINTA01_ALERT_3_C  The value of Rint is greater than 0.12
            Rint given   0.123
PLAT020_ALERT_3_C The Value of Rint is Greater Than 0.12 .........      0.123 Report
PLAT260_ALERT_2_C Large Average Ueq of Residue Including       O97      0.123 Check
PLAT309_ALERT_2_C Single Bonded Oxygen (C-O > 1.3 Ang) ...........        O97 Check
PLAT309_ALERT_2_C Single Bonded Oxygen (C-O > 1.3 Ang) ...........        O17 Check
PLAT309_ALERT_2_C Single Bonded Oxygen (C-O > 1.3 Ang) ...........       O98A Check
PLAT309_ALERT_2_C Single Bonded Oxygen (C-O > 1.3 Ang) ...........       O98B Check
PLAT341_ALERT_3_C Low Bond Precision on  C-C Bonds ...............    0.00729 Ang.
PLAT906_ALERT_3_C Large K Value in the Analysis of Variance ......      2.122 Check
PLAT978_ALERT_2_C Number C-C Bonds with Positive Residual Density.          0 Info
```

Alert level G

```
PLAT002_ALERT_2_G Number of Distance or Angle Restraints on AtSite          7 Note
PLAT004_ALERT_5_G Polymeric Structure Found with Maximum Dimension          3 Info
PLAT007_ALERT_5_G Number of Unrefined Donor-H Atoms ..............          3 Report
PLAT042_ALERT_1_G Calc. and Reported MoietyFormula Strings  Differ     Please Check
PLAT083_ALERT_2_G SHELXL Second Parameter in WGHT  Unusually Large     231.38 Why ?
PLAT172_ALERT_4_G The CIF-Embedded .res File Contains DFIX Records          5 Report
PLAT300_ALERT_4_G Atom Site Occupancy of C97        Constrained at        0.5 Check
PLAT300_ALERT_4_G Atom Site Occupancy of H97A       Constrained at        0.5 Check
PLAT300_ALERT_4_G Atom Site Occupancy of H97B       Constrained at        0.5 Check
PLAT300_ALERT_4_G Atom Site Occupancy of H97C       Constrained at        0.5 Check
PLAT300_ALERT_4_G Atom Site Occupancy of C17B       Constrained at        0.5 Check
PLAT300_ALERT_4_G Atom Site Occupancy of O98A       Constrained at        0.5 Check
PLAT300_ALERT_4_G Atom Site Occupancy of C98A       Constrained at        0.5 Check
PLAT300_ALERT_4_G Atom Site Occupancy of O98B       Constrained at        0.5 Check
PLAT300_ALERT_4_G Atom Site Occupancy of C98B       Constrained at        0.5 Check
PLAT302_ALERT_4_G Anion/Solvent/Minor-Residue Disorder (Resd  2  )        50% Note
PLAT302_ALERT_4_G Anion/Solvent/Minor-Residue Disorder (Resd  3  )        33% Note
PLAT302_ALERT_4_G Anion/Solvent/Minor-Residue Disorder (Resd  4  )       100% Note
PLAT302_ALERT_4_G Anion/Solvent/Minor-Residue Disorder (Resd  5  )       100% Note
PLAT432_ALERT_2_G Short Inter X...Y Contact  O17      ..C97              2.90 Ang.
                                         -1/2+x,1/4-y,7/4-z  =     11_456 Check
PLAT432_ALERT_2_G Short Inter X...Y Contact  O98A     ..C98B             2.00 Ang.
                                             1/2-x,-y,3/2-z  =     25_556 Check
PLAT432_ALERT_2_G Short Inter X...Y Contact  O98A     ..C17B             2.06 Ang.
                                             1/2-x,-y,3/2-z  =     25_556 Check
PLAT432_ALERT_2_G Short Inter X...Y Contact  O98B     ..C17B             1.49 Ang.
                                             1/2-x,-y,3/2-z  =     25_556 Check
PLAT432_ALERT_2_G Short Inter X...Y Contact  O98B     ..C98A             2.38 Ang.
                                             1/2-x,-y,3/2-z  =     25_556 Check
```

```
PLAT432_ALERT_2_G Short Inter X...Y Contact  C17B     ..C98B            2.32 Ang.
                                             1/2-x,-y,3/2-z  =     25_556 Check
PLAT432_ALERT_2_G Short Inter X...Y Contact  C17B     ..C98A            3.09 Ang.
                                                      x,y,z  =      1_555 Check
PLAT432_ALERT_2_G Short Inter X...Y Contact  C17B     ..C98A            3.19 Ang.
                                             1/2-x,-y,3/2-z  =     25_556 Check
PLAT432_ALERT_2_G Short Inter X...Y Contact  C97      ..C97             2.54 Ang.
                                              7/4-x,3/4-y,z  =     14_655 Check
PLAT432_ALERT_2_G Short Inter X...Y Contact  C97      ..C97             2.84 Ang.
                                              x,3/4-y,7/4-z  =      7_556 Check
PLAT432_ALERT_2_G Short Inter X...Y Contact  C98A     ..C98B            0.97 Ang.
                                             1/2-x,-y,3/2-z  =     25_556 Check
PLAT764_ALERT_4_G Overcomplete CIF Bond List Detected (Rep/Expd) .      1.16 Ratio
PLAT794_ALERT_5_G Tentative Bond Valency for Ti1       (IV)      .      4.17 Info
PLAT860_ALERT_3_G Number of Least-Squares Restraints .............         5 Note
PLAT912_ALERT_4_G Missing # of FCF Reflections Above STh/L=  0.600        26 Note
```

---

   0 **ALERT level A** = Most likely a serious problem - resolve or explain
   5 **ALERT level B** = A potentially serious problem, consider carefully
  10 **ALERT level C** = Check. Ensure it is not caused by an omission or oversight
  34 **ALERT level G** = General information/check it is not something unexpected

```
   2 ALERT type 1 CIF construction/syntax error, inconsistent or missing data
  23 ALERT type 2 Indicator that the structure model may be wrong or deficient
   5 ALERT type 3 Indicator that the structure quality may be low
  16 ALERT type 4 Improvement, methodology, query or suggestion
   3 ALERT type 5 Informative message, check
```

Validation response form

Please find below a validation response form (VRF) that can be filled in and pasted into your CIF.

```
# start Validation Reply Form
_vrf_RINTA01_shelx
;
PROBLEM: The value of Rint is greater than 0.12
RESPONSE: ...
;
_vrf_PLAT020_shelx
;
PROBLEM: The Value of Rint is Greater Than 0.12 .........      0.123 Report
RESPONSE: ...
;
_vrf_PLAT260_shelx
;
PROBLEM: Large Average Ueq of Residue Including       O97      0.123 Check
RESPONSE: ...
;
_vrf_PLAT309_shelx
;
PROBLEM: Single Bonded Oxygen (C-O > 1.3 Ang) ...........        O97 Check
RESPONSE: ...
;
_vrf_PLAT341_shelx
;
PROBLEM: Low Bond Precision on  C-C Bonds ...............    0.00729 Ang.
RESPONSE: ...
;
_vrf_PLAT906_shelx
```

```
;
PROBLEM: Large K Value in the Analysis of Variance ......      2.122 Check
RESPONSE: ...
;
_vrf_PLAT978_shelx
;
PROBLEM: Number C-C Bonds with Positive Residual Density.          0 Info
RESPONSE: ...
;
# end Validation Reply Form
```

It is advisable to attempt to resolve as many as possible of the alerts in all categories. Often the minor alerts point to easily fixed oversights, errors and omissions in your CIF or refinement strategy, so attention to these fine details can be worthwhile. In order to resolve some of the more serious problems it may be necessary to carry out additional measurements or structure refinements. However, the purpose of your study may justify the reported deviations and the more serious of these should normally be commented upon in the discussion or experimental section of a paper or in the "special_details" fields of the CIF. checkCIF was carefully designed to identify outliers and unusual parameters, but every test has its limitations and alerts that are not important in a particular case may appear. Conversely, the absence of alerts does not guarantee there are no aspects of the results needing attention. It is up to the individual to critically assess their own results and, if necessary, seek expert advice.

Publication of your CIF in IUCr journals

A basic structural check has been run on your CIF. These basic checks will be run on all CIFs submitted for publication in IUCr journals (*Acta Crystallographica*, *Journal of Applied Crystallography*, *Journal of Synchrotron Radiation*); however, if you intend to submit to *Acta Crystallographica Section C* or *E* or *IUCrData*, you should make sure that full publication checks are run on the final version of your CIF prior to submission.

Publication of your CIF in other journals

Please refer to the *Notes for Authors* of the relevant journal for any special instructions relating to CIF submission.

PLATON version of 19/10/2018; check.def file version of 15/10/2018

Datablock shelx - ellipsoid plot

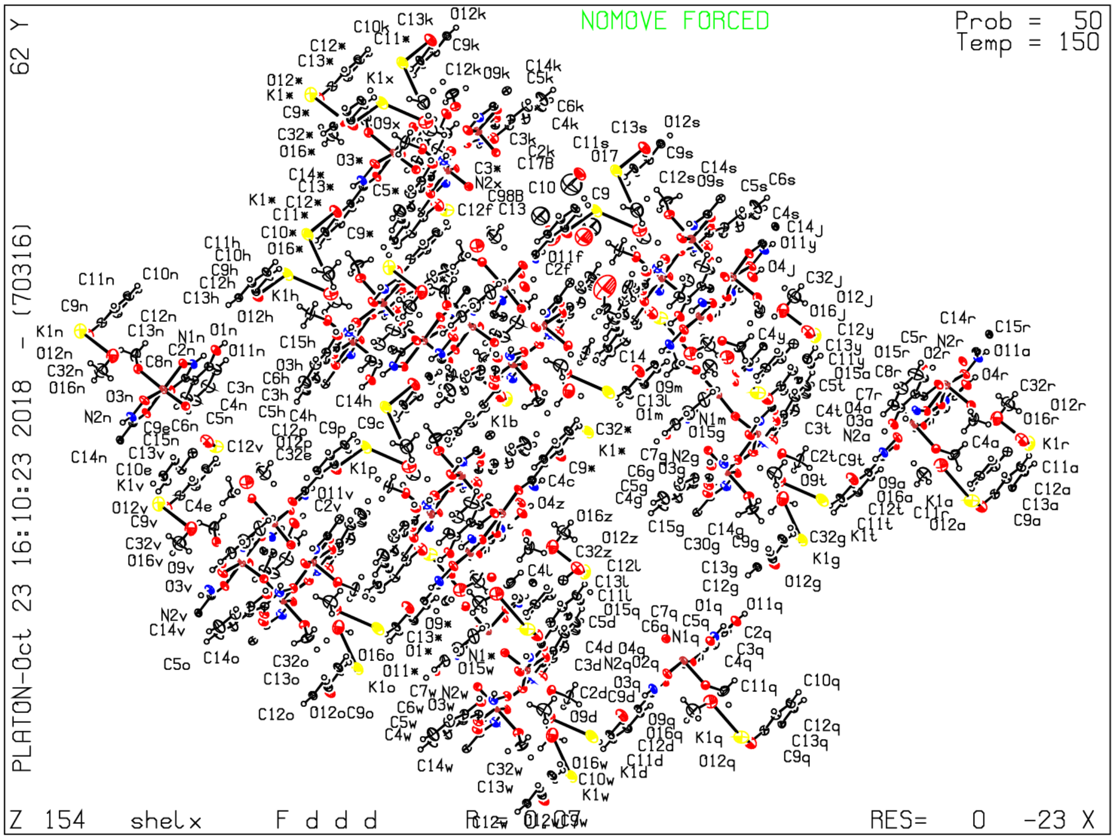

Supplement: Supplementary file 1 [file molecules-26-05588-s001.zip › checkcif_P4.pdf]
