# Supplementary material for: Synthesis, Structural and Physicochemical Characterization of a Titanium(IV) Compound with the Hydroxamate Ligand N,2-Dihydroxybenzamide
Source: Molecules. 2021 Sep 15;26(18):5588. doi: 10.3390/molecules26185588 (PMC8465426; doi:10.3390/molecules26185588)
Supplement: Supplementary file 1 [file molecules-26-05588-s001.zip › Supplementary Materials_PROOFS_HM.pdf]

Supplementary Material: Synthesis, Structural and Physicochemical Characterization of a Titanium(IV) Compound with the Hydroxamate Ligand *N*,2-Dihydroxybenzamide

Stamatis S. Passadis [1], Sofia Hadjithoma [2], Panagiota Siafarika [3], Angelos G. Kalampounias [3,4,] *, Anastasios D. Keramidas [2,*], Haralampos N. Miras, [5,*] and Themistoklis A. Kabanos [1,*]

Contents

Figure S1. Possible modes for hydroxamate binding S2

Figure S2. Experimental and calculated IR spectra for the H_2dihybe and **1**. S2

Figure S3. 2D {^{1}H,^{13}C} HMBC of H_2dihybe S3

Figure S4. 2D {^{1}H} grCOSY of **1** S4

Figure S5. 2D {^{1}H,^{13}C} grHSQC of **1** S5

Figure S6. 2D {^{1}H,^{13}C} grHMBC of **1** S6

Figure S7. 2D {^{1}H,^{13}C} grHSQC of **1** + 4 eq But_4NOH + 0.1 eq H_2dihybe S7

Figure S8. 2D {^{1}H,^{13}C} grHMBC of **1** + 4 eq But_4NOH + 0.1 eq H_2dihybe S8

Figure S9. Tauc plot of compound **1** S9

Table S1. ^{1}H and ^{13}C NMR of H_3dihybe and species **2** S10

Table S2. ^{1}H and ^{13}C NMR of H_2dihybe and **1** after addition But_4NOH S11

References S12

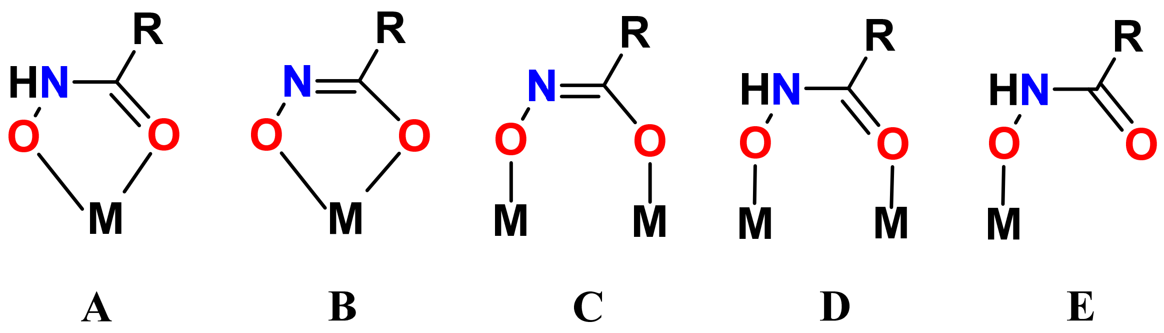

Figure S1. Possible modes for hydroxamate binding.

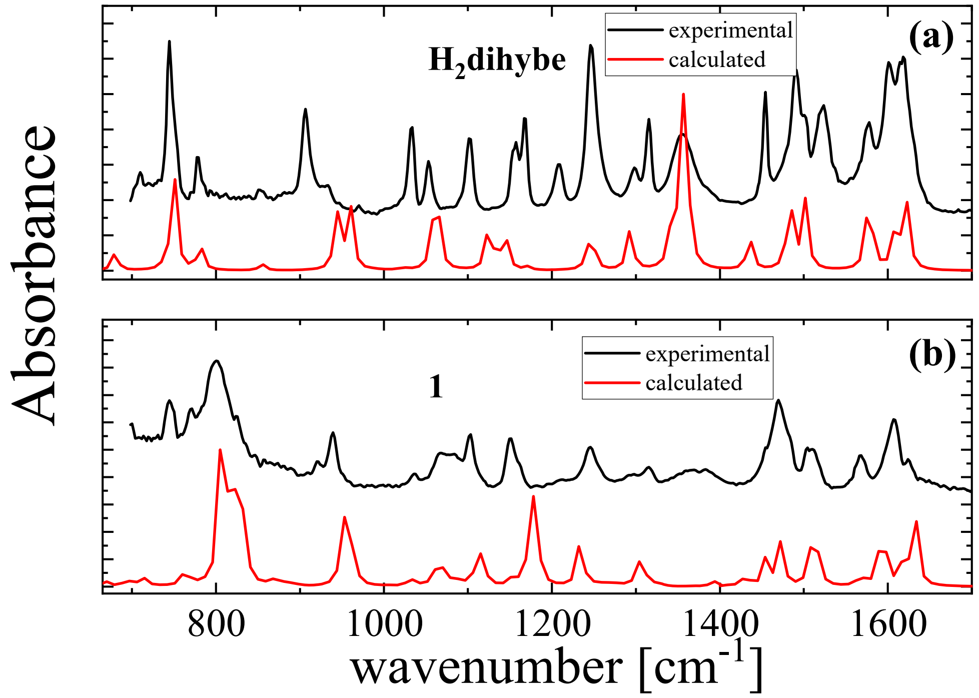

Figure S2. Experimental and calculated in vacuum by ab initio DFT/B3LYP/LANL2DZ modeling spectra for the H_2dihybe (a) and **1** (b).

In Figures S2(a) and S2(b) are shown the experimental and theoretically predicted IR absorbance spectra of the H_2dihybe and Ti-complex 1, respectively. It seems that for both cases, namely for the H_2dihybe and for the 1, all main bands observed in the experimental spectra are also present in the calculated spectra which is in good agreement with the proposed structural model. Possible differences in their relative intensities and frequency shifts, mainly in the low-frequency region, are reasonable

considering that the calculation was performed in the vapor state without the presence of any additional interactions. The theoretical calculation performed to establish the formation of the Ti-complex in the solid state.

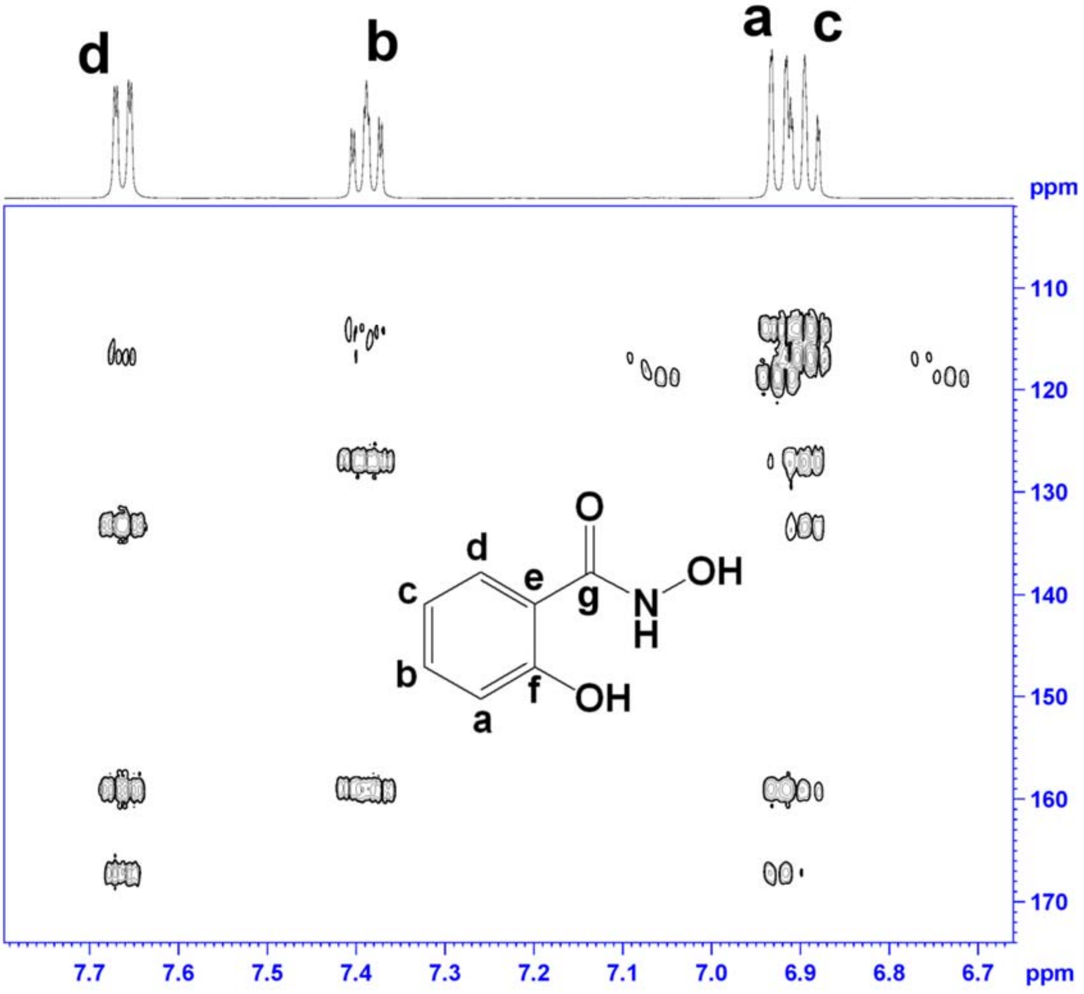

Figure S3. 2D {^{1}H,^{13}C} HMBC of H_2dihybe.

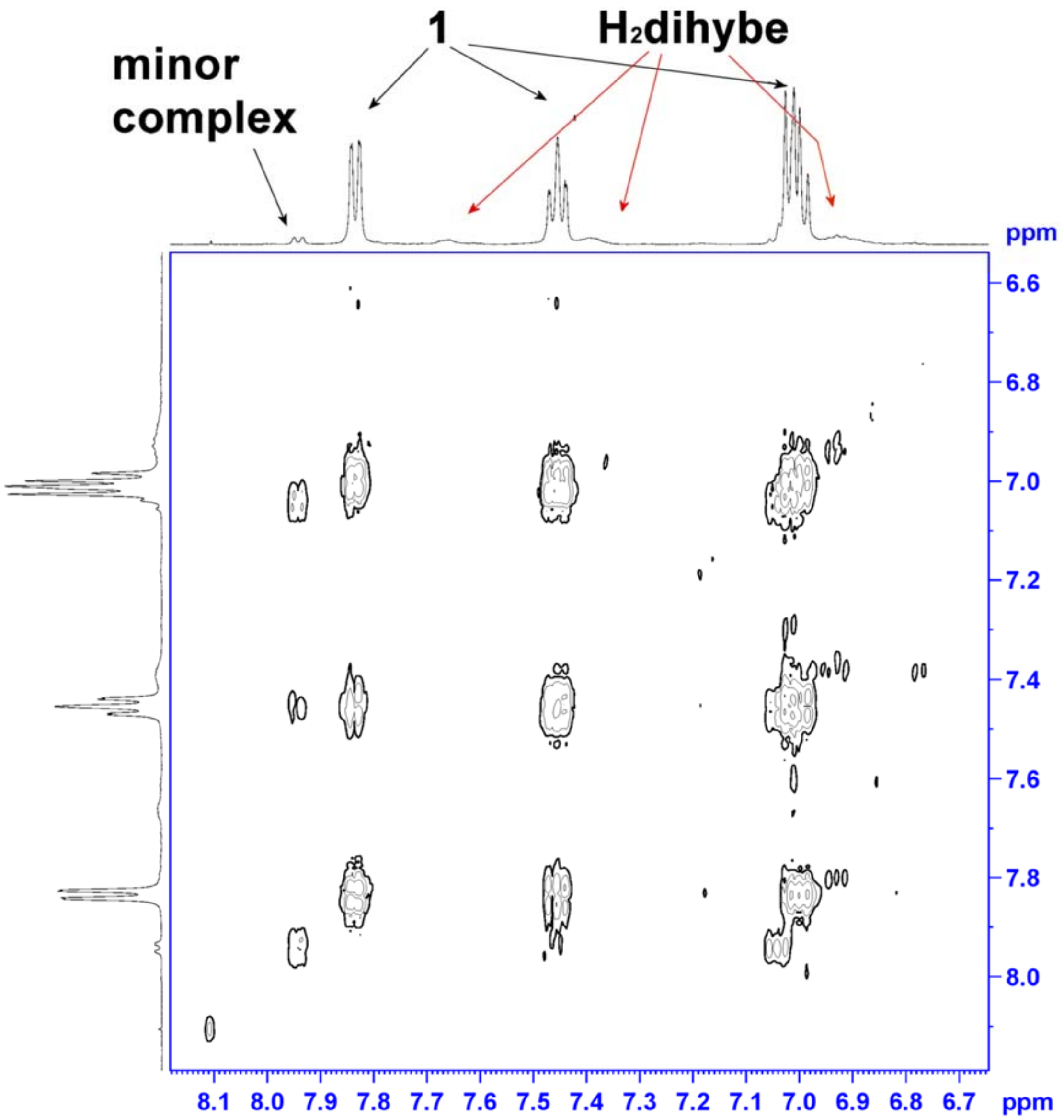

Figure S4. 2D {^{1}H} grCOSY of **1**.

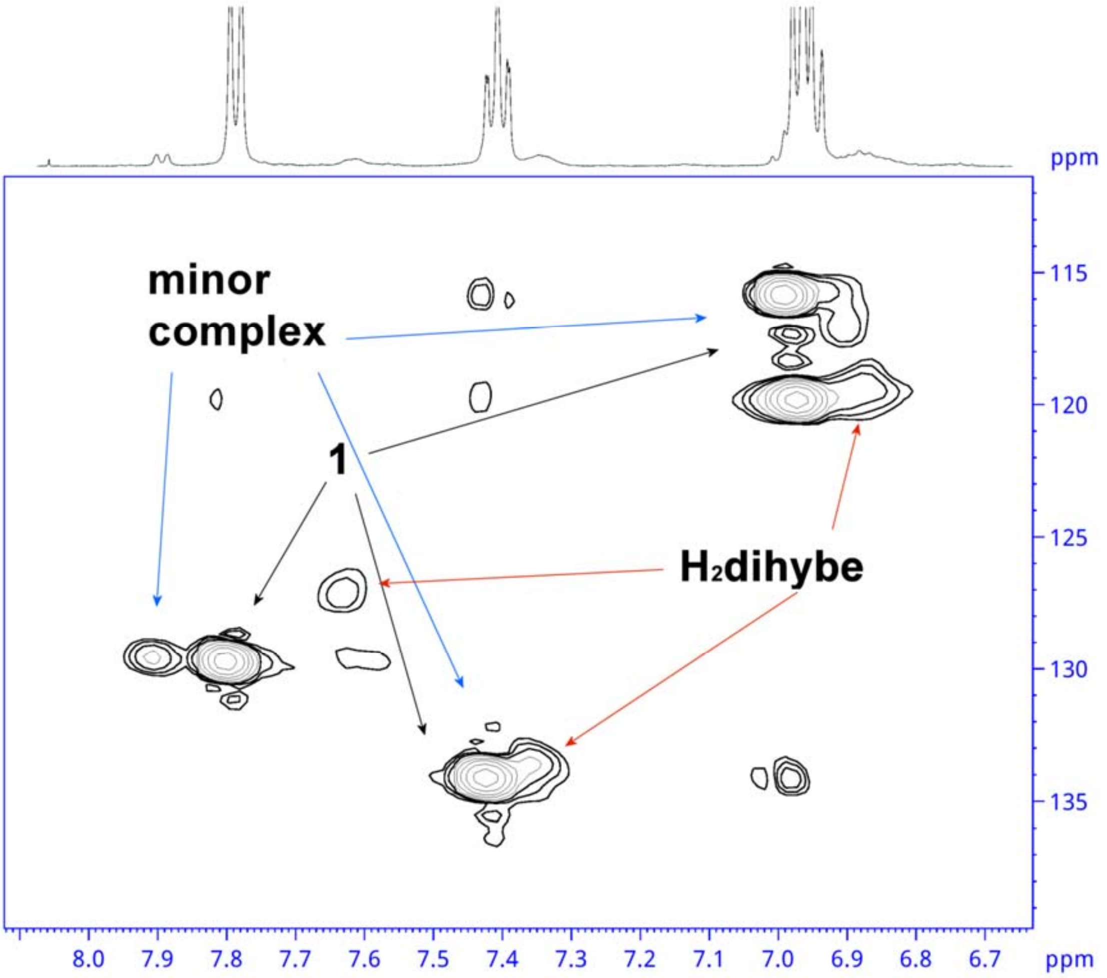

Figure S5. 2D {^{1}H,^{13}C} grHSQC of **1**.

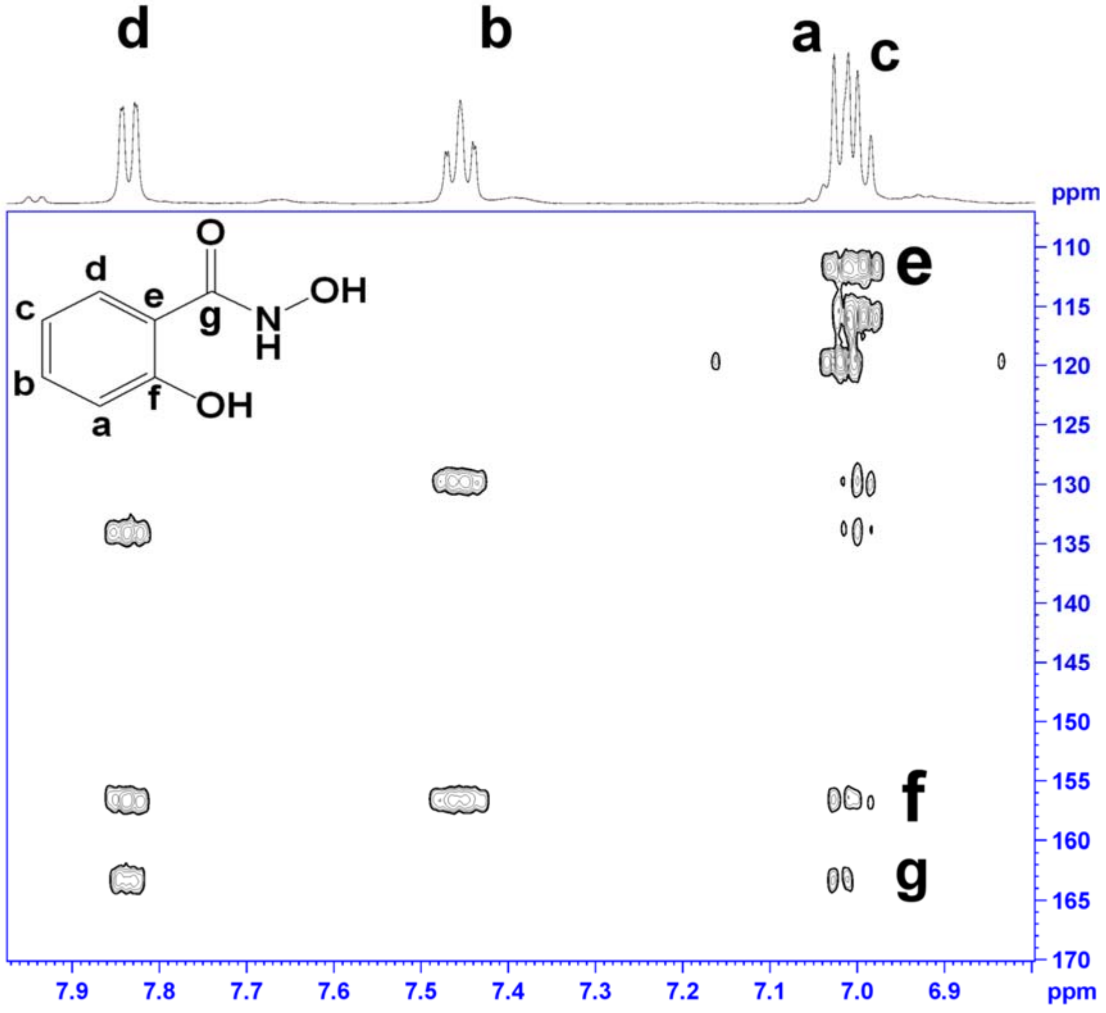

Figure S6. 2D {^{1}H,^{13}C} grHMBC of **1**.

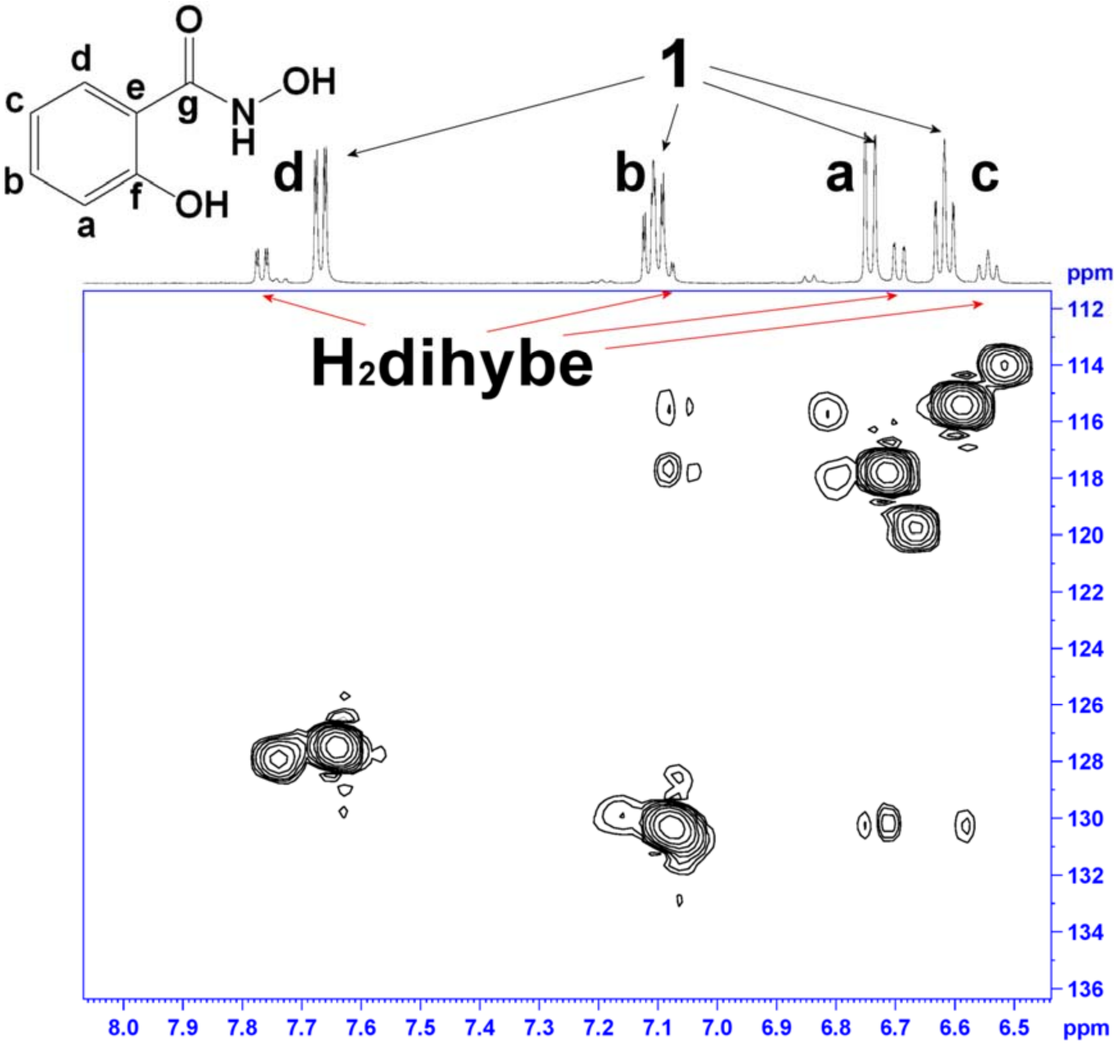

Figure S7. 2D {^{1}H,^{13}C} grHSQC of **1** + 4 eq But_4NOH + 0.1 eq H_2dihybe.

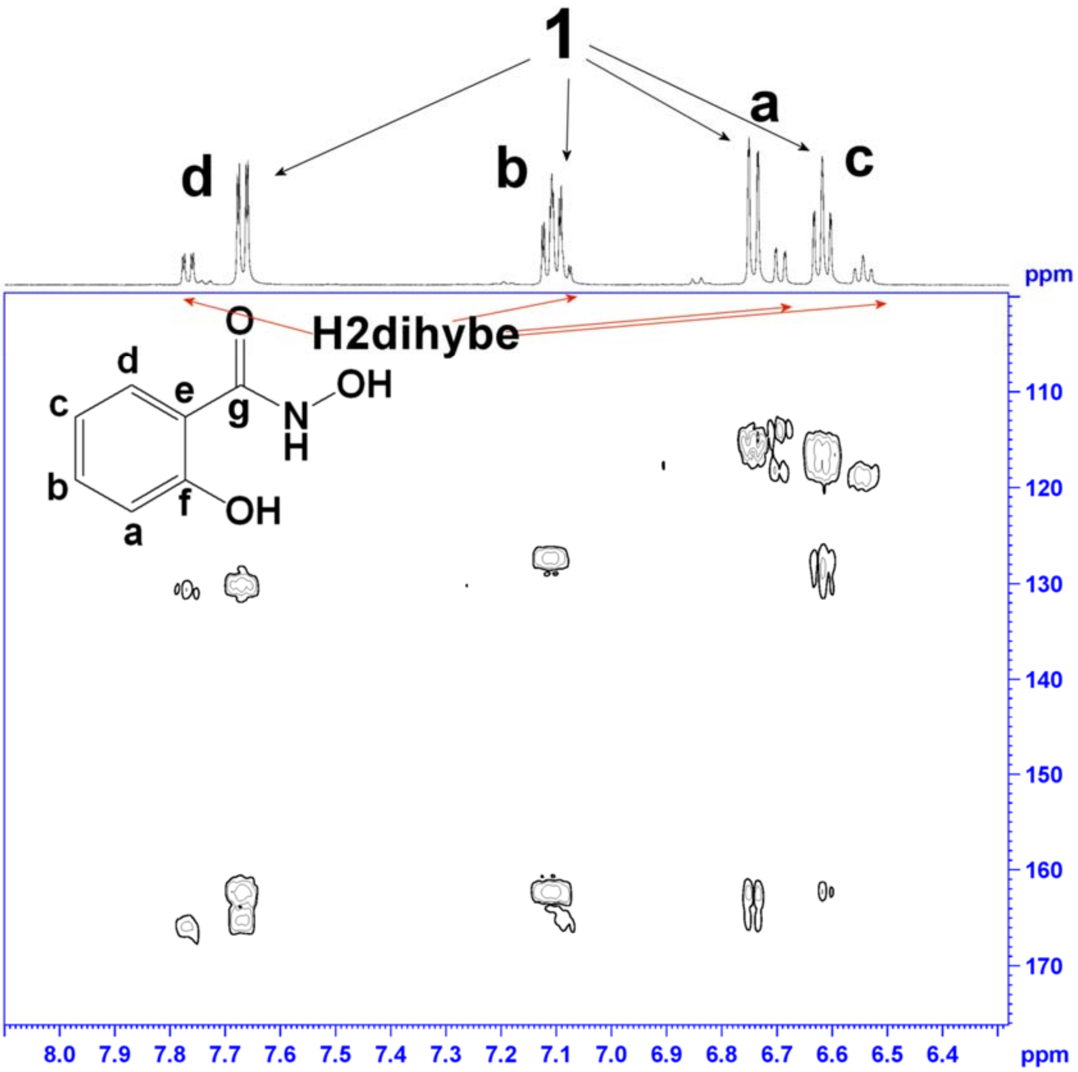

Figure S8. 2D {^{1}H,^{13}C} grHMBC of **1** + 4 eq But_4NOH + 0.1 eq H_2dihybe.

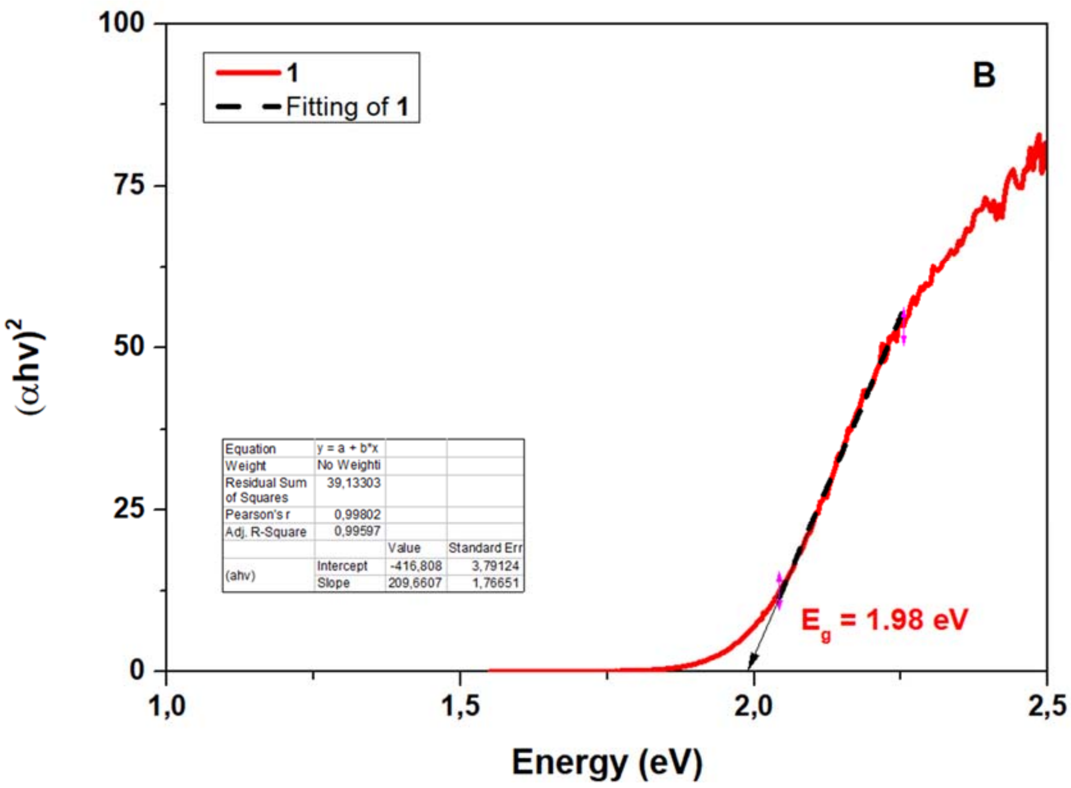

Figure S9. Tauc plot of compound **1**.

Table S1. ^{1}H and ^{13}C NMR chemical shifts (ppm) for the ligand H_3dihybe, the minor titanium species **2** and the shielding/deshielding effect ($\Delta\delta$) upon complexation.$^{\alpha}$

	**2**		H_2dihybe			
	**^{13}C**	**^{1}H**	**^{13}C**	**^{1}H**	**^{13}C($\Delta\delta$,ppm)$^{\beta}$**	**^{1}H($\Delta\delta$,ppm)$^{\beta}$**
**C(d)$^{\gamma}$**	129.7	7.947	128.2	7.669	1.5	0.278
**C(c)$^{\gamma}$**	119.9	7.004	119.4	6.897	0.5	0.107
**C(b)$^{\gamma}$**	134.3	7.436	133.6	7.338	0.7	0.098
**C(a)$^{\gamma}$**	115.5	7.017	117.2	6.928	-1.7	0.089
**C(g)$^{\gamma}$**			167.2			
**C(f)$^{\gamma}$**			159.2			
**C(e)$^{\gamma}$**			114.1			

$^{\alpha}$ The chemical shifts of the protons are the center of multiplets. $^{\beta}$ $\Delta\delta$ is the chemical shift difference between the chemical shift of the NMR peaks (^{13}C or ^{1}H) of the complex **2** and the respective peaks of the ligand. $^{\gamma}$ The carbon atoms of the ligand H_2dihybe shown in Figure 6.

Table S2. ^{1}H and ^{13}C NMR chemical shifts (ppm) for **1** and H$_2$dihybe after addition of three equivalents of But$_4$NOH per **1**[a] and the shielding/deshielding effect ($\Delta\delta$, ppm) upon complexation.

	**1**		**H$_2$dihybe**			
	**^{13}C**	**^{1}H**	**^{13}C**	**^{1}H**	**^{13}C($\Delta\delta$,ppm)$^{\beta}$**	**^{1}H($\Delta\delta$,ppm)$^{\alpha,\beta}$**
**C(d)**$^{\gamma}$	127.6	7.667	128.0	7.779	-0.4	-0.112
**C(c)**$^{\gamma}$	115.4	6.617	114.1	6.457	1.3	0.160
**C(b)**$^{\gamma}$	130.4	7.017	131.6	7.021	-1.2	-0.004
**C(a)**$^{\gamma}$	117.9	6.740	119.8	6.657	-1.9	0.083
**C(g)**$^{\gamma}$	165.2		165.9		-0.7	
**C(f)**$^{\gamma}$	162.5		165.9		-0.4	
**C(e)**$^{\gamma}$	116.3		118.4		-2.1	

$^{\alpha}$ The chemical shifts for the protons are the center of multiplets. $^{\beta}$ $\Delta\delta$ is the chemical shift difference between the chemical shift of the NMR peaks (either ^{13}C or ^{1}H) of the complex **1** and the respective peaks of the ligand. $^{\gamma}$ The carbon atoms of the ligand H$_2$dihybe shown in Figure 6.

Addition of one to four equivalents But$_4$NOH to the CD$_3$OD solution of **1** and/or H$_2$dihybe, results in a shift of the aromatic proton and carbon peaks (Table S2, Figure 6D) compared to the free ligand, presumably due to deprotonation of the phenolic oxygen atom (Table S2)

In addition, the ^{1}H NMR peaks of **1** with base are much sharper than the peaks of **1** without base, suggesting that after the addition of the base the chemical exchange is slower and the coordination of the ligand to the titanium is stronger. The values of ^{13}C ($\Delta\delta$, ppm) from -0.4 to -2.1 (Table S2), suggest a similar ligation of the ligand to Ti^{IV}

through the carbonylate and hydroxamate oxygen atoms. Coordination of Ti^{IV} to the deprotonated phenolic oxygen atom is excluded, because the $\Delta\delta$ (ppm) value of -0.4 for **^{13}C(d)** is much smaller than the expected (6 to 8 ppm) [1-5].

References

1. Passadis, S. S.; Papanikolaou, M. G.; Elliott, A.; Tsiafoulis, C. G.; Tsipis, A. C.; Keramidas, A. D.; Miras, H. N.; Kabanos, T. A. Synthesis, Structural, and Physicochemical Characterization of a Ti_6 and a Unique Type of Zr_6 Oxo Clusters Bearing an Electron-Rich Unsymmetrical {OON} Catecholate/Oxime Ligand and Exhibiting Metalloaromaticity. *Inorg. Chem.* **2020**, *59*, 18345-18357.
2. Drouza, C.; Hadjithoma, S.; Nicolaou, M.; Keramidas, A. D. Structural characterization, hydrolytic stability, and dynamics of cis-$Mo^{VI}O_2^{2+}$ hydroquinonate/phenolate complexes. *Polyhedron.* **2018**, *152*, 22-30.
3. Drouza, C.; Stylianou, M.; Keramidas, A. D., NMR characterization and dynamics of vanadium(V) complexes with tripod (hydroquinonate/phenolate) iminodiacetate ligands in aqueous solution. *Pure Appl. Chem.* **2009**, *81* (7), 1313-1321.
4. Drouza, C.; Stylianou, M.; Papaphilippou, P.; Keramidas, A. D., Structural and electron paramagnetic resonance (EPR) characterization of novel vanadium(V/IV) complexes with hydroquinonate-iminodiacetate ligands exhibiting "noninnocent" activity. *Pure Appl. Chem.* **2013**, *85* (2), 329-342.
5. Passadis, S. S.; Hadjithoma, S.; Kalampounias, A. G.; Tsipis, A. C.; Sproules, S.; Miras, H. N.; Keramidas, A. D.; Kabanos, T. A., Synthesis, structural and

physicochemical characterization of a new type Ti6-oxo cluster protected by a cyclic imide dioxime ligand. *Dalton Trans.* **2019**, *48* (17), 5551-5559.
